# Successful risk stratification of a patient with ventricular preexcitation by improved transesophageal electrophysiological study

**DOI:** 10.1111/anec.12882

**Published:** 2021-07-21

**Authors:** Chao Qin, Tao He, Shuo Li

**Affiliations:** ^1^ Department of ECG Diagnosis First Affiliated Hospital of Guangxi Medical University Nanning China

**Keywords:** atrial fibrillation, risk stratification, transesophageal electrophysiological study, ventricular fibrillation, ventricular preexcitation

## Abstract

The patient is a 19 years‐old man who often wakes up in dreams with palpitations and fatigue. The ECG shows: 1. Sinus rhythm; 2. Preexcitation syndrome. Transesophageal electrophysiological study (TEEPS) diagnosis:High‐risk accessory pathway. During radiofrequency catheter ablation, the patient suddenly developed atrial fibrillation and quickly converted to ventricular fibrillation. After defibrillation, ventricular fibrillation is transformed into sinus rhythm. Subsequently, the patient's high‐risk accessory pathway was successfully ablated. Studies have shown that about 25% of patients with WPW syndrome have a refractory period of less than 250 ms, which is one of the risk factors for the conversion of atrial fibrillation to ventricular fibrillation. Therefore, risk stratification is recommended for these symptomatic patients. From 1980 to 1990, there were literature reports on risk stratification of patients with preexcitation syndrome by TEEPS. But it has not become a routine examination of risk stratification in patients with preexcitation syndrome.The reason may be related to the hardware conditions and risk stratification methods used at that time. The TEEPS equipment currently used in our hospital can control the pacing voltage at about 12 mv on average. The voltage in this case report is 9 mv only. In addition, we successfully stratified the risk of patient with preexcitation syndrome without inducing atrial fibrillation. All the electrophysiological records of the patient during the examination were recorded simultaneously with the 12‐lead ECG and the esophageal lead ECG. These improvements makes TEEPS a simple, safe and reliable non‐invasive cardiac electrophysiological detection technology, which is worth popularizing in hospitals.

A 19‐year‐old man without structural heart disease presented with recurrent episodes of parodical palpitations during sleep. He was examined by a regular 12‐lead electrocardiogram(ECG) in the local hospital, and the results showed the following: 1. sinus rhythm and 2. ventricular preexcitation. After inhaling oxygen, the symptoms were improved. For further diagnosis and treatment, the patient came to our hospital for transesophageal electrophysiology studies (TEEPS) (Figure 1).

**FIGURE 1:**
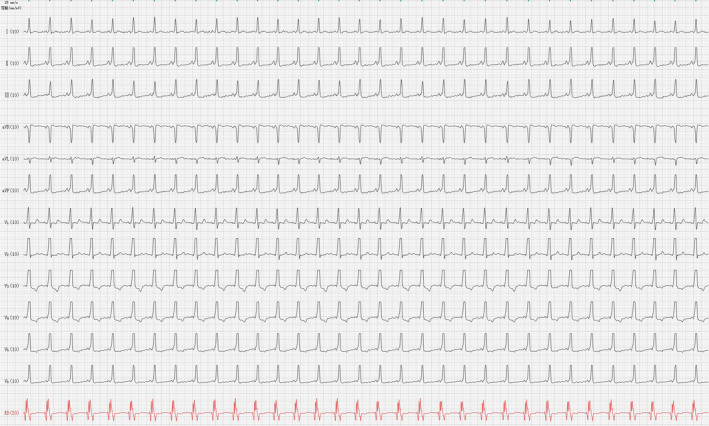
Esophageal ECG shows ventricular preexcitation

The electrophysiological characteristics of TEEPS are as follows: When the S1S1 stimulation frequency is set at 260bpm, the atrioventricular conduction still shows a descending ratio of 1:1 (Figure 2).

**FIGURE 2:**
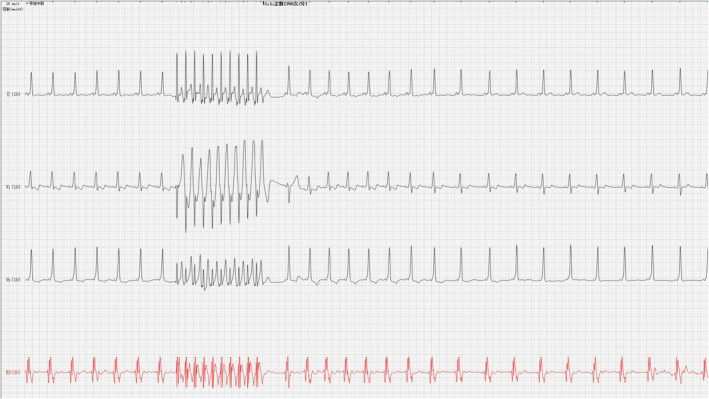
S1S1 260bpm, 1:1 atrioventricular conduction

When the basic perimeter of S_1_S_2_ stimulation is set to 500–220 ms, we can observe that the S_2_ stimulation pulse successfully activates the atrium and descends to the ventricle, and the QRS waveform shows a complete preexcitation pattern. When the retrograde scan continued to 210 ms, the patient's atrium entered the refractory period, and no QRS wave was seen after S_2_ stimulation (Figure 3). Supraventricular tachycardia was not induced during the whole process, and there was no obvious atrioventricular node double pathway jump. TEEPS suggests high‐risk accessory pathways.

**FIGURE 3:**
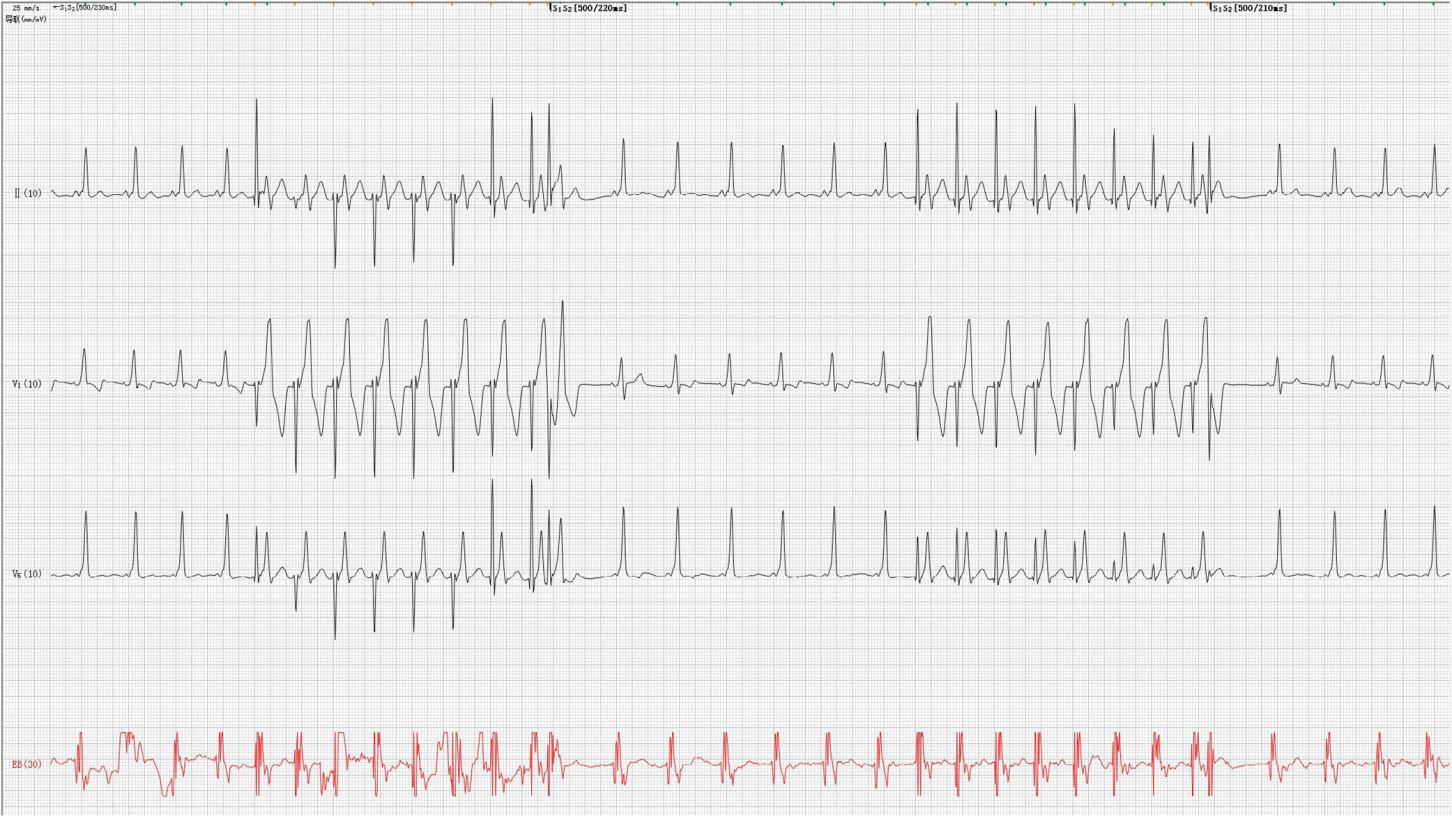
S1S2 500ms‐220ms, atrial activation descending to ventricle via accessory pathway

The patient underwent radiofrequency catheter ablation. According to the results of TEEPS, operator has taken corresponding safety measures in advance to deal with sudden malignant arrhythmias.

During the operation, the patient suddenly developed a wide QRS tachycardia (Figure 4); the heart rate was more than 300 beats/min, and the RR interval was irregular. It was diagnosed as atrial fibrillation (AF), and then, the rapid AF quickly turned into ventricular fibrillation (VF) (Figure 5, Figure 6). At this time, the patient's blood pressure was 90/60 mmHg, and hemodynamic instability such as convulsion and loss of consciousness appeared. The operator immediately defibrillated and successfully converted VF into sinus rhythm. Then, the patient's high‐risk bypass was successfully ablated.

**FIGURE 4:**
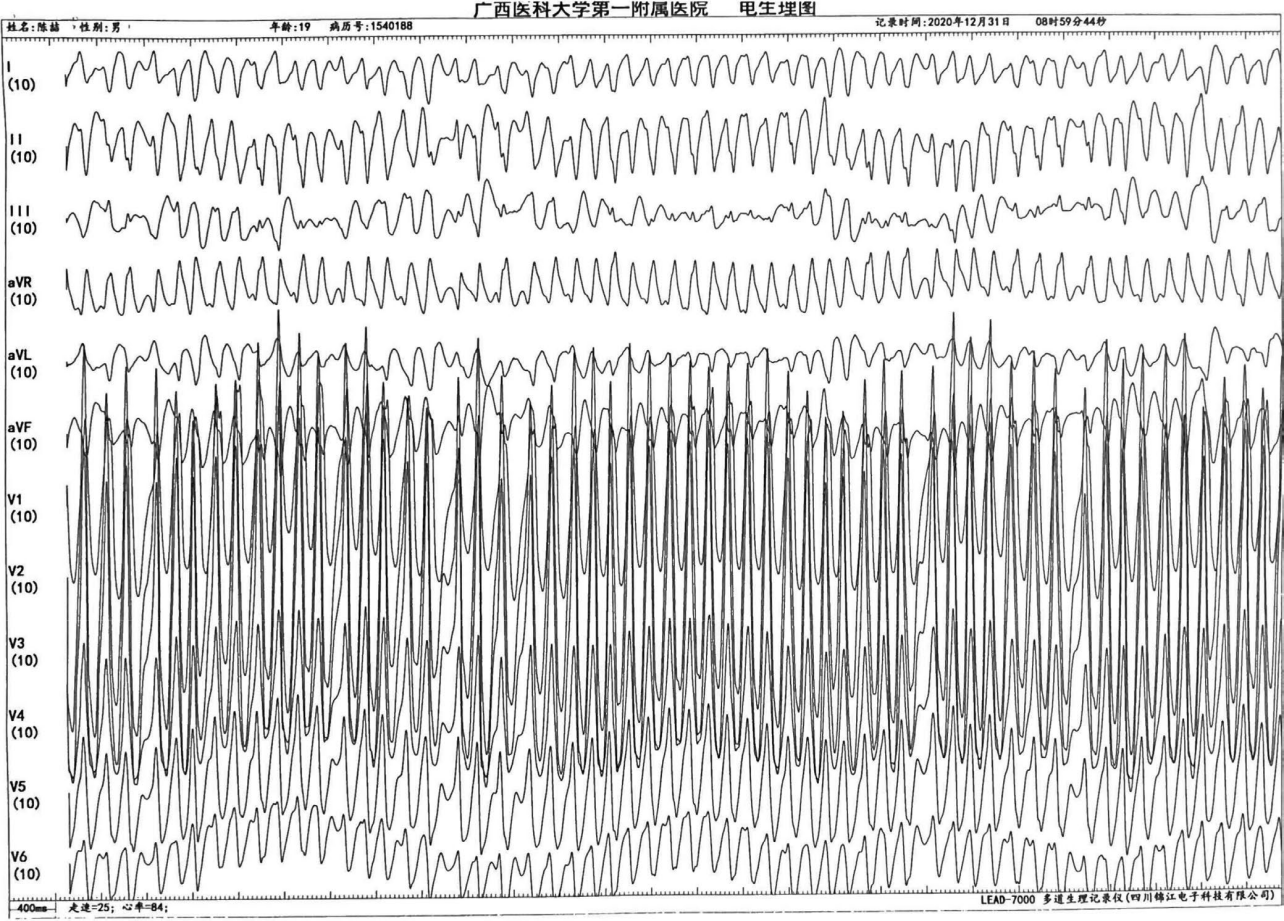
AF with ventricular preexcitation

**FIGURE 5:**
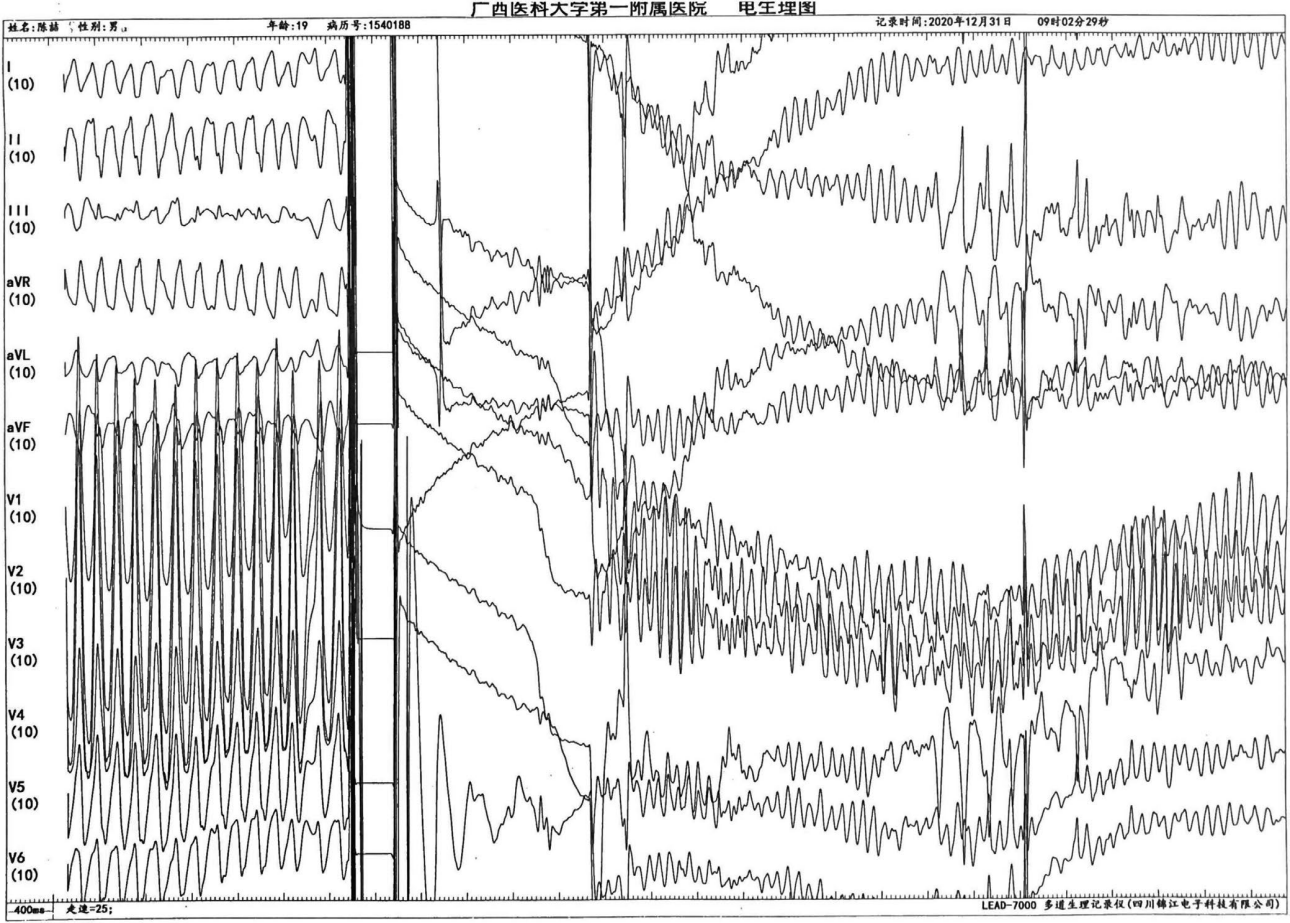
AF transformed into VF

**FIGURE 6:**
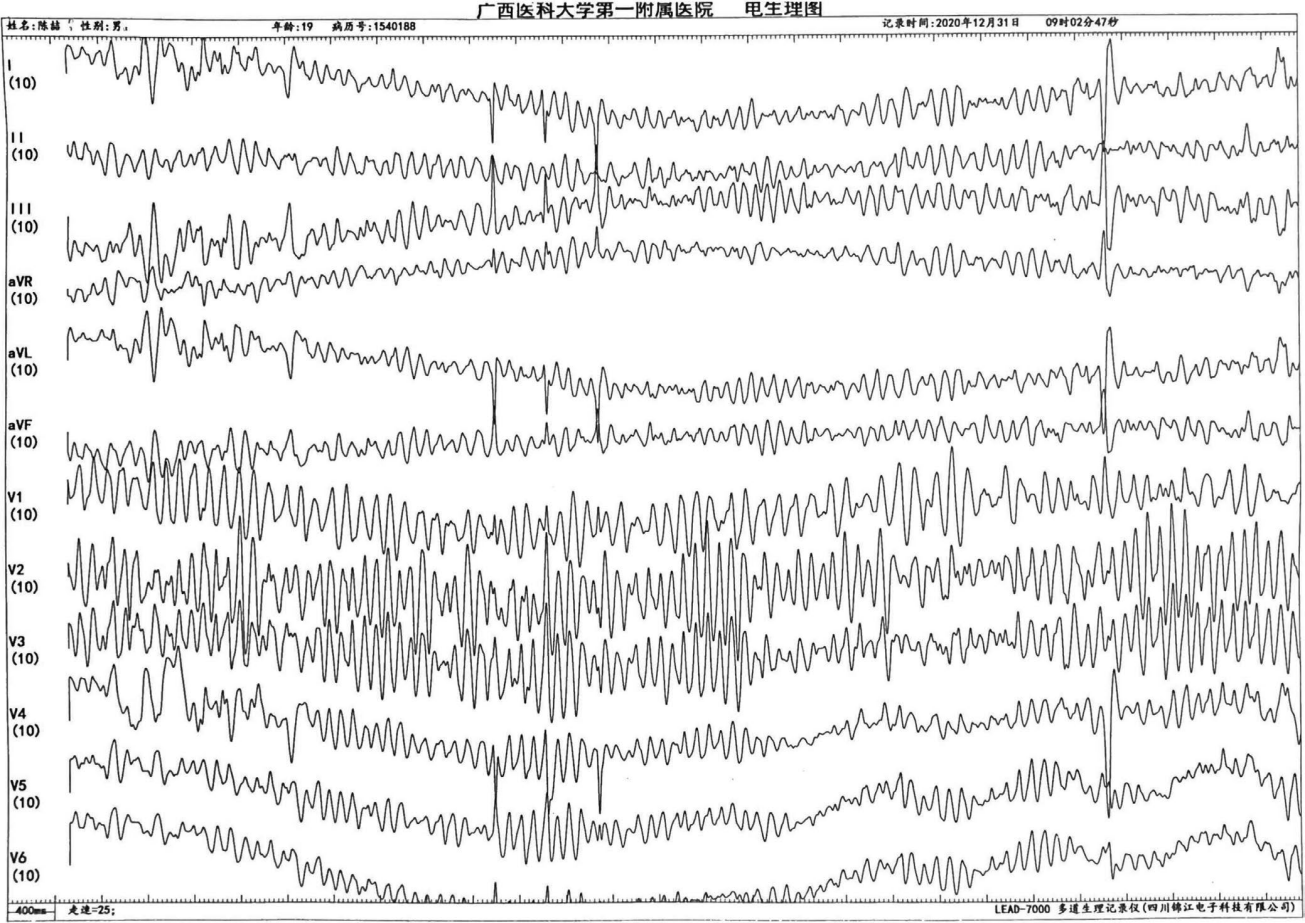
Ventricular fibrillation

The ECG of the patient was reexamined after operation (Figure 7): 1, sinus rhythm and 2, elevation of ST segment (suggesting early repolarization). Three days after operation, the patient had no obvious discomfort, and all the examination indexes were normal and were discharged from the hospital.

**FIGURE 7:**
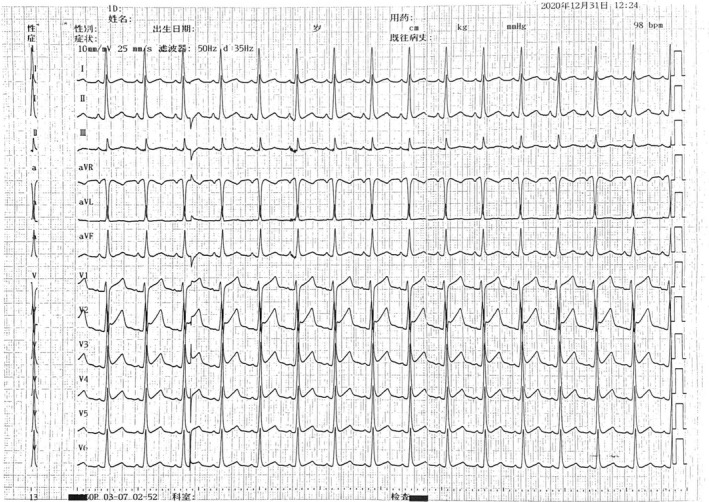
Sinus rhythm with elevation of ST segment (hint of early repolarization)

According to statistics, the proportion of AF in ventricular preexcitation is about 15%, but the mechanism is still unclear (Pietersen et al., 1992). Studies have shown that the antegrade refractory period of accessory pathway in about 25% of patients with ventricular preexcitation is less than 250 ms, which is a risk factor for the progression of AF to VF (Zardini et al., 1994). Ventricular preexcitation affects about 0.1%–0.3% of the general population (Hiss & Lamb, 1962). When ventricular preexcitation is accompanied by syncope or palpitation, it can be diagnosed as Wolff–Parkinson–White (WPW) syndrome (Munger et al., 1993; Wolff et al.,). Patients with WPW syndrome have an increased risk of sudden cardiac death, which may be close to 4% in their lifetime (Al‐Khatib & Pritchett, 1999). Therefore, it is recommended to stratify the risk of these symptomatic patients (Blomström‐Lundqvist et al., 2003). Non‐invasive risk stratification methods include the following: Holter, exercise stress test, and TEEPS. The predictive value of the first two is relatively low (Koca et al., 2017), but the effects of TEEPS and invasive intracardiac electrophysiology are similar (Toni & Blaufox, 2012). However, the hospitalization cost, invasiveness, and risk of intracardiac electrophysiological examination are relatively increased. In contrast, TEEPS is easier to perform because of its lower cost, less risk, and higher success rate (Hoyt et al., 2013a). In this case, TEEPS showed that the QRS wave was still transmitted through the accessory pathway in S_1_S_2_ 500–220 ms, and the atrium entered the refractory period in S_1_S_2_ 500–210 ms. Therefore, the real refractory period of accessory pathway is less than 210 MS, suggesting high‐risk accessory pathway. Because the measurement of antegrade refractory period of accessory pathway is easily affected by atrial refractory period, some researchers believe that measuring the shortest RR interval of AF induced by intracardiac electrophysiology is a reliable index to predict high‐risk preexcitation patients, one literature reported the use of adenosine to increase the chance of inducing atrial fibrillation during TEEPS to enable risk stratification in asymptomatic patients with ventricular preexcitation(Hoyt et al., 2013b). Although this method is relatively effective, it also has the risk of causing atrial fibrillation and then developing into ventricular fibrillation. TEEPS is to determine the related parameters and induce specific types of PSVT by pacing atrium. If the accessory pathway's refractory period is shorter than the atrioventricular node's refractory period, it will be disadvantageous to the formation of antegrade reentrant loop. This is one of the reasons why PSVT was not successfully induced during pacing. During PSVT attacks, due to the short accessory pathway refractory period and fast conduction velocity, the ventricular rate of patients is faster than normal, resulting in increased intratribal pressure, atrial ischemia, and electrophysiological instability. With the increase of atrial vulnerability caused by many factors, the probability of inducing AF is also increased, and there is a risk of developing VF (Gallagher et al., 1978). Therefore, when the patient's high‐risk bypass is clearly diagnosed, it is recommended to end the TEEPS immediately and not to force the induction of PSVT. If patients with ventricular preexcitation are routinely examined by TEEPS before operation, the risk of ventricular preexcitation can be stratified, to clearly diagnose whether there is a high‐risk bypass and provide a strong basis for the follow‐up diagnosis and treatment of patients. From 1980 to 1990, there were literature reports on risk stratification of patients with preexcitation syndrome by TEEPS. But it has not become a routine examination of risk stratification in patients with preexcitation syndrome. The reason may be related to the hardware conditions and risk stratification methods used at that time. For example, excessive pacing voltage makes patients feel painful during the examination. It has also been reported in the literature that it is necessary to induce atrial fibrillation before measuring RR interval in order to carry out risk stratification. In contrast, the TEEPS equipment currently used in our hospital can control the pacing voltage at about 12 mv on average. The voltage in this case report is 9 mv only. In addition, we successfully stratified the risk of patient with preexcitation syndrome without inducing atrial fibrillation. All the electrophysiological records of the patient during the examination were recorded simultaneously with the 12‐lead ECG and the esophageal lead ECG. These improvements make TEEPS a simple, safe, and reliable non‐invasive cardiac electrophysiological detection technology, which is worth popularizing in hospitals.

## CONFLICT OF INTEREST

The authors have no competing interests, funding, or financial relationships to disclose.

## Author Contributions

Chao Qin is mainly responsible for the TEEP examination of this patient and the writing of the case report. Tao He is mainly responsible for the supervision and guidance of TEEP and the communication of article publication. Shuo Li is mainly responsible for tracking the patient's follow‐up electrophysiological examination results and collecting the recorded pictures of radiofrequency ablation surgery.

## Ethical Approval

The authors declare that they have no conflict of interest. All procedures performed in studies involving human participants were in accordance with the ethical standards of the institutional and/or national research committee and with the 1964 Helsinki declaration and its later amendments or comparable ethical standards. This article does not contain any studies with animals performed by any of the authors. Informed consent was obtained from all individual participants included in the study.

## Data Availability

Data sharing not applicable to this article as no datasets were generated or analysed during the current study.
